# Supplementary material for: Effects of RIPC on the Metabolome in Patients Undergoing Vascular Surgery: A Randomized Controlled Trial
Source: Biomolecules. 2022 Sep 16;12(9):1312. doi: 10.3390/biom12091312 (PMC9496371; doi:10.3390/biom12091312)
Supplement: Supplementary file 1 [file biomolecules-12-01312-s001.zip › Table S1.pdf]

Table S1. Baseline comparison of the analyzed metabolites between the groups

Metabolite	Group						*p*-value
	Sham			RIPC			
	Mean/ Median	SD/ Q1	Q3	Mean/ median	SD/ Q1	Q3	
**Ala**	392.47	91.42		385.53	89.88		0.715
**Arg**	114.26	29.25		120.41	35.37		0.365
**Cit**	35.85	8.24		34.20	9.77		0.384
**Gln**	811.96	132.49		836.51	186.13		0.470
**Glu***	72.90	55.40	95.90	57.10	46.40	74.80	0.036
**Gly***	240.00	189.00	286.00	247.00	202.00	288.00	0.885
**His**	91.83	19.36		94.65	18.72		0.479
**Ile***	85.00	72.80	103.00	85.50	70.60	108.00	0.867
**Leu***	180.00	147.00	203.00	168.00	144.00	205.00	0.680
**Lys**	251.85	62.25		271.04	70.97		0.171
**Met**	22.98	5.60		24.48	6.39		0.232
**Orn**	96.26	25.91		97.40	23.49		0.826
**Phe***	70.50	63.70	83.30	72.10	65.80	78.30	0.697
**Pro**	204.74	49.65		206.58	63.30		0.877
**Ser**	134.02	34.93		133.09	31.36		0.893
**Thr***	147.00	109.00	251.00	111.00	92.45	161.00	0.072
**Trp***	64.90	54.30	76.40	66.00	53.90	74.40	0.885
**Tyr**	64.45	12.72		70.94	15.07		0.028
**Val**	266.49	53.09		265.51	71.65		0.941
**ADMA**	0.56	0.15		0.55	0.14		0.741
**Creatinine***	105.00	81.30	151.00	108.00	76.40	143.00	0.640
**Kynurenine***	0.04	0.03	0.05	0.04	0.03	0.05	0.353
**Serotonine***	0.43	0.33	0.62	0.51	0.34	0.66	0.421
**Spermine***	0.00	0.00	3.84	0.00	0.00	3.84	0.711
**Taurine**	107.03	29.92		105.62	32.00		0.827
**Total DMA**	1.12	0.29		1.06	0.29		0.330
**lysoPCaC16:0***	117.00	78.00	238.00	104.00	77.30	235.00	0.895
**lysoPCaC16:1***	3.49	2.35	6.29	3.29	2.44	6.29	0.935
**lysoPCaC17:0***	2.11	1.40	3.99	1.85	1.22	4.47	0.678
**lysoPCaC18:0**	22.52	7.14		22.74	7.34		0.881
**lysoPCaC18:1***	25.00	19.00	44.40	26.45	17.00	41.70	0.696
**lysoPCaC18:2***	35.80	21.70	50.00	39.30	20.70	57.80	0.551
**lysoPCaC20:3***	2.21	1.62	3.43	2.03	1.52	4.51	0.854
**lysoPCaC20:4***	7.29	5.44	13.10	6.73	4.55	12.80	0.501
**lysoPCaC26:1**	0.63	0.40		0.74	0.57		0.360
**PCaaC28:1***	2.59	2.12	3.05	2.77	2.05	3.26	0.618
**PCaaC30:0***	3.04	2.51	4.01	3.03	2.78	3.69	0.864
**PCaaC32:0***	11.00	7.98	15.30	12.30	7.36	17.10	0.806
**PCaaC32:1***	13.60	8.05	21.10	13.60	9.15	22.20	0.815
**PCaaC32:2**	2.38	1.59		2.26	1.46		0.715
**PCaaC32:3***	0.36	0.22	0.50	0.31	0.24	0.47	0.641
**PCaaC34:1***	242.00	209.00	314.00	262.00	211.00	317.00	0.953
**PCaaC34:2**	338.43	129.76		345.13	129.30		0.805
**PCaaC34:4***	0.98	0.48	1.58	0.81	0.51	1.25	0.614

**PCaaC36:0***	2.21	1.65	2.77	1.99	1.52	2.70	0.268
**PCaaC36:1**	37.71	15.26		40.66	17.02		0.383
**PCaaC36:2**	188.63	60.04		196.90	62.22		0.518
**PCaaC36:3**	102.03	39.23		103.89	35.60		0.812
**PCaaC36:4**	165.60	64.69		160.29	60.36		0.685
**PCaaC36:5***	27.80	16.60	39.80	22.95	16.85	36.85	0.800
**PCaaC38:0**	2.75	0.93		2.48	0.66		0.119
**PCaaC38:3**	39.95	13.38		40.40	12.81		0.869
**PCaaC38:4***	90.80	66.90	107.00	81.60	66.50	119.00	0.885
**PCaaC38:5**	48.49	17.49		47.08	14.83		0.677
**PCaaC38:6***	79.20	61.50	108.00	76.20	66.50	98.60	0.700
**PCaaC40:4**	2.56	0.65		2.66	0.83		0.540
**PCaaC40:5**	7.75	2.42		7.89	2.57		0.780
**PCaaC40:6***	28.90	22.80	38.20	30.90	24.20	36.80	0.994
**PCaaC42:4**	0.17	0.04		0.16	0.04		0.267
**PCaaC42:5**	0.34	0.10		0.32	0.08		0.299
**PCaaC42:6**	0.53	0.17		0.58	0.15		0.221
**PCaeC30:1***	0.45	0.32	0.57	0.37	0.26	0.65	0.737
**PCaeC32:1***	1.93	1.54	2.94	2.03	1.44	2.93	0.861
**PCaeC32:2***	0.56	0.44	0.77	0.59	0.43	0.75	1.000
**PCaeC34:0***	1.06	0.85	1.46	1.04	0.87	1.36	0.978
**PCaeC34:1**	8.04	3.12		7.96	3.35		0.909
**PCaeC34:2**	7.21	2.93		7.23	2.97		0.979
**PCaeC34:3***	3.73	2.64	5.44	3.65	2.31	5.91	0.815
**PCaeC36:0**	0.85	0.25		0.85	0.31		0.978
**PCaeC36:1**	6.76	2.52		6.56	2.32		0.701
**PCaeC36:2**	10.20	3.49		10.02	3.50		0.811
**PCaeC36:3***	4.56	3.70	6.34	4.62	3.68	6.55	0.885
**PCaeC36:4***	11.20	8.05	14.00	9.77	7.91	13.60	0.441
**PCaeC36:5***	7.47	6.29	10.50	7.21	5.46	10.60	0.419
**PCaeC38:0**	1.74	0.71		1.68	0.59		0.650
**PCaeC38:3**	3.23	0.96		3.14	0.90		0.655
**PCaeC38:4**	9.51	2.43		9.03	2.02		0.316
**PCaeC38:5**	13.05	3.13		12.34	3.68		0.323
**PCaeC38:6***	5.59	4.43	7.46	5.19	4.20	7.32	0.349
**PCaeC40:1**	1.23	0.35		1.33	0.42		0.268
**PCaeC40:2***	1.70	1.44	1.93	1.59	1.43	1.97	0.584
**PCaeC40:4***	1.86	1.61	2.22	1.65	1.52	1.97	0.104
**PCaeC40:5**	2.86	0.62		2.66	0.55		0.114
**PCaeC40:6***	4.05	3.45	4.91	3.79	3.34	4.36	0.222
**PCaeC42:4**	0.70	0.19		0.67	0.19		0.483
**PCaeC44:4***	0.40	0.30	0.46	0.32	0.26	0.45	0.649
**PCaeC44:5***	1.37	1.06	1.80	1.29	1.09	1.66	0.499
**PCaeC44:6**	0.99	0.40		0.89	0.23		0.174
**SM(OH)C14:1***	3.22	2.28	5.14	2.96	2.30	4.93	0.779
**SM(OH)C16:1***	1.74	1.14	2.60	1.53	1.16	2.62	0.750
**SM(OH)C22:1***	6.01	4.69	8.60	6.40	4.89	7.72	0.755
**SM(OH)C22:2***	5.38	4.33	6.94	5.05	4.22	6.98	0.686
**SM(OH)C24:1**	0.70	0.29		0.69	0.19		0.744
**SMC16:0***	66.50	41.80	94.00	71.00	36.70	90.90	0.594

**SMC16:1***	8.80	6.51	12.70	8.87	5.83	12.50	0.573
**SMC18:0***	21.50	16.00	31.70	21.70	16.40	26.60	0.950
**SMC18:1***	5.71	4.28	8.82	6.13	3.65	8.56	0.610
**SMC20:2***	0.18	0.12	0.29	0.18	0.12	0.21	0.559
**SMC24:0**	11.17	4.21		11.18	3.54		0.993
**SMC24:1**	34.55	9.76		33.01	8.85		0.431
**SMC26:0***	0.09	0.08	0.14	0.10	0.08	0.13	0.667
**SMC26:1**	0.30	0.11		0.31	0.11		0.841
**H1***	4825.00	4245.00	5231.00	4505.00	4207.00	5013.00	0.128
**(C2+C3)/C0**	0.24	0.08		0.21	0.06		0.105
**AAA**	204.23	35.65		211.71	36.80		0.325
**ADMA/Arg***	0.01	0.00	0.01	0.01	0.00	0.01	0.841
**BCAA**	532.19	96.74		537.89	143.34		0.825
**C2/C0***	0.18	0.16	0.24	0.18	0.15	0.22	0.320
**Cit/Arg**	0.34	0.12		0.31	0.13		0.276
**Cit/Orn**	0.39	0.11		0.37	0.12		0.342
**Essential AA**	1087.47	208.40		1081.53	212.42		0.893
**Fisher ratio**	2.64	0.44		2.54	0.50		0.326
**Glucogenic AA***	740.00	690.00	872.00	765.00	701.00	839.00	0.770
**Kynurenine/Trp**	0.04	0.02		0.04	0.01		0.654
**Nonessential AA**	2319.57	252.78		2346.04	338.07		0.671
**Orn/Arg***	0.78	0.66	1.06	0.83	0.66	1.02	0.741
**Putrescine/Orn***	0.00	0.00	0.00	0.00	0.00	0.00	0.595
**Serotonin/Trp***	0.01	0.01	0.01	0.01	0.01	0.01	0.391
**Total SM**	180.96	59.56		169.40	49.07		0.346
**Total SM-nonOH**	158.61	53.58		148.05	44.12		0.326
**Total SM-OH***	17.40	13.30	23.80	15.80	13.40	23.70	0.898
**Total SM-OH/Total SM-nonOH**	0.12	0.03		0.12	0.02		0.542
**Tyr/Phe**	0.91	0.20		0.98	0.21		0.115

All metabolites are measured in μmol/L, except for metabolic ratios, which do not have a unit
* Non-normal distribution (Kolmogorov-Smirnov's test). In the case of a non-normal distribution, median and quartiles (Q1, Q3) are provided. In the case of a normal distribution, mean and standard deviation (SD) are given. SD - standard deviation, Q1 - first quartile, Q3 - third quartile, Ala - Alanine, Arg - Arginine, Cit - Citrulline, Gln - Glutamine, Glu - Glutamic acid, Gly - Glycine, His - Histidine, Ile - Isoleucine, Leu - Leucine, Lys - Lysine, Met - Methionine, Orn - Ornithine, Phe - Phenylalanine, Pro - Proline, Ser - Serine, Thr - Threonine, Trp - Tryptophan, Tyr - Tyrosine, Val - Valine, ADMA - Asymmetric dimethylarginine, DMA - dimethylarginine, lysoPCa - lysoPhosphatidylcholine acyl, PCaa - Phosphatidylcholine diacyl, PCae - Phosphatidylcholine acyl-alkyl, SM(OH) - Hydroxysphingomyeline, SM - Sphingomyeline, H1 - hexose, C2 - Acetylcarnitine, C3 - Propionylcarnitine, C0 - Carnitine, AAA - Aminoadipic acid, ADMA - asymmetric dimethylarg-inine, BCAA – Branched chain amino acids, AA - Amino acids, OH - hydroxy, nonOH – Nonhydroxy
