# Supplementary material for: Effects of RIPC on the Metabolome in Patients Undergoing Vascular Surgery: A Randomized Controlled Trial
Source: Biomolecules. 2022 Sep 16;12(9):1312. doi: 10.3390/biom12091312 (PMC9496371; doi:10.3390/biom12091312)
Supplement: Supplementary file 1 [file biomolecules-12-01312-s001.zip › Table S2.pdf]

Table S2. Change in the metabolites 24 h after operation, comparison between the groups

Metabolite	Group						p-value
	Sham			RIPC			
	Mean/ median	SD/ Q1	Q3	Mean/ median	SD/ Q1	Q3	
**Ala**	-25.26	120.74		-11.56	123.72		0.592
**Arg***	-20.00	-38.70	-4.20	-21.00	-35.80	-2.00	0.591
**Cit**	-8.83	8.94		-7.63	7.77		0.496
**Gln**	-150.49	169.93		-161.47	189.39		0.770
**Glu***	-0.70	-15.50	14.60	-12.70	-24.00	14.00	0.128
**Gly**	-26.32	53.43		-22.38	52.73		0.723
**His**	-12.69	13.42		-14.72	18.57		0.550
**Ile**	-16.43	28.88		-24.95	32.72		0.188
**Leu**	-28.18	54.57		-46.08	52.25		0.112
**Lys**	-50.32	65.53		-56.52	63.24		0.645
**Met**	-1.63	7.26		-1.23	9.58		0.822
**Orn**	-25.24	30.58		-26.01	24.84		0.894
**Phe**	2.21	12.68		-1.08	14.84		0.255
**Pro***	-13.00	-55.00	26.00	-17.00	-42.00	21.00	0.666
**Ser***	-27.10	-37.90	-14.00	-32.00	-53.80	-10.00	0.222
**Thr***	-21.60	-49.05	18.00	-20.90	-81.00	12.00	0.516
**Trp**	-8.81	17.38		-10.01	15.16		0.725
**Tyr**	-3.87	16.12		-5.33	18.89		0.692
**Val**	-22.24	74.64		-37.78	72.87		0.315
**ADMA**	-0.09	0.18		-0.08	0.18		0.833
**Creatinine***	1.00	-9.00	20.00	-3.70	-15.00	22.00	0.322
**Kynurenine**	0.06	0.74		0.09	0.97		0.842
**Serotonine**	-0.06	0.13		-0.07	0.15		0.717
**Spermine***	0.00	0.00	0.00	0.00	0.00	0.00	0.030
**Taurine**	-14.88	30.13		-10.28	31.39		0.475
**Total DMA**	-0.08	0.34		-0.09	0.37		0.932
**lysoPCaC16:0***	-41.30	-96.00	-25.40	-43.00	-99.00	-26.80	0.643
**lysoPCaC16:1***	-1.12	-2.03	-0.66	-1.20	-3.30	-0.65	0.309
**lysoPCaC17:0***	-0.81	-1.84	-0.38	-0.93	-2.06	-0.46	0.376
**lysoPCaC18:0***	-9.90	-13.68	-7.28	-9.96	-16.07	-6.00	0.873
**lysoPCaC18:1***	-12.18	-19.25	-5.69	-11.80	-25.20	-7.93	0.717
**lysoPCaC18:2***	-21.00	-34.10	-5.90	-15.00	-32.20	-6.40	0.833
**lysoPCaC20:3***	-0.94	-1.94	-0.62	-0.96	-1.61	-0.53	0.885
**lysoPCaC20:4***	-2.41	-4.50	-1.10	-2.82	-5.54	-1.56	0.513
**lysoPCaC26:1***	-0.05	-0.14	0.08	-0.03	-0.12	0.06	0.670
**PCaaC28:1***	-0.46	-0.81	-0.07	-0.36	-0.71	-0.08	0.399
**PCaaC30:0***	-0.77	-1.27	-0.26	-0.69	-1.04	-0.40	0.791
**PCaaC32:0**	-1.17	2.81		-1.68	1.94		0.321
**PCaaC32:1***	-1.90	-4.40	-0.22	-1.80	-5.53	-0.16	0.938
**PCaaC32:2***	-0.46	-1.20	-0.16	-0.63	-1.13	-0.26	0.483
**PCaaC32:3***	-0.06	-0.09	-0.01	-0.05	-0.08	0.00	0.840
**PCaaC34:1**	-26.40	58.84		-33.38	47.97		0.536
**PCaaC34:2**	-39.81	75.21		-45.71	50.00		0.657
**PCaaC34:4***	-0.24	-0.36	-0.11	-0.23	-0.54	-0.10	0.536

**PCaaC36:0**	-0.47	0.54		-0.48	0.40		0.956
**PCaaC36:1***	-7.20	-13.50	-4.70	-5.90	-12.10	-2.90	0.264
**PCaaC36:2**	-39.09	37.01		-44.61	29.63		0.433
**PCaaC36:3***	-20.40	-29.00	-12.00	-16.00	-29.00	-3.30	0.240
**PCaaC36:4**	-15.06	42.18		-20.88	25.73		0.425
**PCaaC36:5***	-4.30	-7.45	-1.85	-2.35	-11.20	-0.34	0.335
**PCaaC38:0***	-0.47	-0.74	-0.26	-0.39	-0.84	-0.12	0.489
**PCaaC38:3**	-8.33	8.60		-9.88	7.56		0.363
**PCaaC38:4**	-12.55	19.84		-17.05	12.61		0.196
**PCaaC38:5**	-8.79	11.96		-9.94	7.83		0.585
**PCaaC38:6***	-11.80	-17.70	-3.00	-7.30	-18.50	2.60	0.126
**PCaaC40:4**	-0.49	0.53		-0.66	0.56		0.139
**PCaaC40:5**	-1.47	1.66		-1.79	1.38		0.322
**PCaaC40:6***	-5.30	-8.40	-2.20	-3.80	-7.00	-0.60	0.264
**PCaaC42:4**	-0.03	0.04		-0.02	0.04		0.315
**PCaaC42:5**	-0.07	0.08		-0.06	0.07		0.612
**PCaaC42:6***	-0.12	-0.17	-0.03	-0.08	-0.15	0.00	0.233
**PCaeC30:1***	0.03	-0.16	0.13	0.01	-0.13	0.10	0.893
**PCaeC32:1**	-0.39	0.48		-0.42	0.33		0.722
**PCaeC32:2***	-0.09	-0.18	-0.04	-0.08	-0.14	-0.03	0.643
**PCaeC34:0**	-0.24	0.21		-0.23	0.22		0.741
**PCaeC34:1**	-1.38	1.65		-1.36	1.46		0.944
**PCaeC34:2**	-1.86	1.27		-1.91	1.42		0.850
**PCaeC34:3**	-1.22	0.89		-1.31	1.09		0.641
**PCaeC36:0**	-0.07	0.18		-0.11	0.17		0.360
**PCaeC36:1***	-0.86	-1.40	-0.46	-0.68	-1.96	-0.26	0.904
**PCaeC36:2***	-1.70	-2.89	-1.03	-1.60	-2.59	-0.79	0.646
**PCaeC36:3***	-1.29	-1.96	-0.80	-1.15	-1.72	-0.57	0.559
**PCaeC36:4**	-2.32	1.92		-2.63	1.99		0.450
**PCaeC36:5**	-1.79	1.55		-1.96	1.51		0.597
**PCaeC38:0***	-0.41	-0.58	-0.17	-0.35	-0.70	-0.13	0.632
**PCaeC38:3**	-0.54	0.66		-0.61	0.61		0.605
**PCaeC38:4**	-1.46	1.81		-1.79	1.17		0.297
**PCaeC38:5**	-2.39	2.34		-2.74	1.89		0.444
**PCaeC38:6**	-1.28	1.16		-1.33	0.83		0.787
**PCaeC40:1**	-0.27	0.24		-0.31	0.36		0.546
**PCaeC40:2**	-0.26	0.35		-0.33	0.27		0.308
**PCaeC40:4***	-0.30	-0.44	-0.20	-0.25	-0.57	0.02	0.365
**PCaeC40:5**	-0.42	0.52		-0.47	0.36		0.636
**PCaeC40:6**	-0.69	0.84		-0.82	0.57		0.377
**PCaeC42:4**	-0.11	0.15		-0.15	0.15		0.252
**PCaeC44:4**	-0.05	0.09		-0.07	0.13		0.490
**PCaeC44:5***	-0.21	-0.28	-0.10	-0.21	-0.34	-0.02	0.957
**PCaeC44:6***	-0.16	-0.24	-0.07	-0.12	-0.25	-0.03	0.264
**SM(OH)C14:1***	-0.57	-1.05	-0.26	-0.47	-0.84	-0.11	0.240
**SM(OH)C16:1**	-0.20	0.42		-0.26	0.28		0.368
**SM(OH)C22:1***	-1.10	-1.92	-0.55	-0.89	-1.93	-0.30	0.481
**SM(OH)C22:2**	-1.00	1.27		-1.06	0.85		0.761
**SM(OH)C24:1**	-0.14	0.18		-0.13	0.15		0.805
**SMC16:0**	-11.77	13.46		-11.64	10.45		0.960

**SMC16:1**	-1.13	1.90		-1.17	1.19		0.904
**SMC18:0**	-2.81	4.99		-3.23	4.60		0.680
**SMC18:1**	-0.50	1.35		-0.70	1.12		0.453
**SMC20:2***	-0.03	-0.05	0.00	-0.02	-0.06	0.02	0.849
**SMC24:0**	-2.11	2.25		-2.52	1.74		0.338
**SMC24:1**	-5.10	7.35		-6.08	5.64		0.475
**SMC26:0**	-0.01	0.05		-0.01	0.05		0.901
**SMC26:1**	-0.04	0.09		-0.06	0.07		0.414
**H1**	1231.64	1798.12		1157.69	1431.53		0.828
**(C2+C3)/C0**	-0.05	0.07		-0.04	0.08		0.744
**AAA**	-10.40	37.01		-16.33	38.51		0.453
**ADMA/Arg***	0.00	0.00	0.00	0.00	0.00	0.00	0.776
**BCAA***	-104.00	-183.00	-29.00	-81.00	-159.00	53.00	0.238
**C2/C0**	-0.04	0.06		-0.04	0.07		0.564
**Cit/Arg**	-0.02	0.11		0.00	0.11		0.422
**Cit/Orn***	0.04	-0.07	0.09	0.00	-0.12	0.14	0.474
**Essential AA**	-159.98	254.73		-185.36	241.36		0.625
**Fisher ratio**	-0.24	0.56		-0.32	0.56		0.456
**Glucogenic AA**	-84.81	162.93		-57.76	175.56		0.445
**Kynurenine/Trp***	0.01	0.00	0.01	0.01	0.00	0.02	0.741
**Nonessential AA**	-339.91	362.31		-324.04	413.17		0.845
**Orn/Arg***	-0.10	-0.24	0.08	-0.12	-0.25	0.08	0.901
**Putrescine/Orn***	0.00	0.00	0.00	0.00	0.00	0.00	0.413
**Serotonin/Trp***	0.00	0.00	0.00	0.00	0.00	0.00	0.991
**Total SM**	-27.53	34.38		-30.78	24.52		0.624
**Total SM-nonOH**	-23.88	30.22		-25.88	22.34		0.731
**Total SM-OH**	-3.09	3.69		-3.46	2.72		0.583
**Total SM-OH/Total SM-nonOH**	0.00	0.01		0.00	0.01		0.578
**Tyr/Phe**	-0.09	0.18		-0.07	0.22		0.692

All metabolites are measured in μmol/L, except for metabolic ratios, which do not have a unit

* Non-normal distribution (Kolmogorov-Smirnov's test). In the case of a non-normal distribution, median and quartiles (Q1, Q3) are provided. In the case of a normal distribution, mean and standard deviation (SD) are given. SD - standard deviation, Q1 - first quartile, Q3 - third quartile, Ala - Alanine, Arg - Arginine, Cit - Citrulline, Gln - Glutamine, Glu - Glutamic acid, Gly - Glycine, His - Histidine, Ile - Isoleucine, Leu - Leucine, Lys - Lysine, Met - Methionine, Orn - Ornithine, Phe - Phenylalanine, Pro - Proline, Ser - Serine, Thr - Threonine, Trp - Tryptophan, Tyr - Tyrosine, Val - Valine, ADMA - Asymmetric dimethylarginine, DMA - dimethylarginine, lysoPCa - lysoPhosphatidylcholine acyl, PCaa - Phosphatidylcholine diacyl, PCae - Phosphatidylcholine acyl-alkyl, SM(OH) - Hydroxysphingomyeline, SM - Sphingomyeline, H1 - hexose, C2 - Acetylcarnitine, C3 - Propionylcarnitine, C0 - Carnitine, AAA - Aminoadipic acid, ADMA - asymmetric dimethylarg-inine, BCAA – Branched chain amino acids, AA - Amino acids, OH - hydroxy, nonOH – Nonhydroxy
